# Effect of hyperhomocysteinemia on the prognostic value of triglyceride glucose index in patients with acute coronary syndrome

**DOI:** 10.3389/fcvm.2024.1517437

**Published:** 2025-01-10

**Authors:** Yi Kan, Xiaoteng Ma, Zehao Zhao, Shutong Dong, Yinxiao Xu, Yan Sun, Yujing Cheng, Dai Zhang, Yuyang Liu, Xiaoli Liu, Dongmei Shi, Yujie Zhou

**Affiliations:** Beijing Key Laboratory of Precision Medicine of Coronary Atherosclerotic Disease, Department of Cardiology, Beijing Anzhen Hospital, Beijing Institute of Heart Lung and Blood Vessel Disease, Clinical Center for Coronary Heart Disease, Capital Medical University, Beijing, China

**Keywords:** hyperhomocysteinemia, triglyceride glucose index, acute coronary syndrome, percutaneous coronary intervention, major adverse cardiovascular events

## Abstract

**Background:**

The prognostic value of triglyceride-glucose (TyG) has been well described in patients with coronary artery disease (CAD). Hyperhomocysteinemia (HHcy) promotes insulin resistance and has also been regarded as a potential risk factor for cardiovascular disease. However, the prognostic value of TyG in acute coronary syndrome (ACS) patients undergoing percutaneous coronary intervention (PCI) and the interaction between TyG and HHcy remain unclear.

**Methods:**

A total of 1,734 ACS patients undergoing PCI were continuously enrolled between June 2016 and November 2017 at Beijing Anzhen Hospital. Patients were categorized into four groups based on HHcy status and the optimal cut-off value of TyG. The primary endpoint was major adverse cardiovascular events (MACE), a composite of all-cause death, nonfatal myocardial infarction, nonfatal stroke, and unplanned repeat revascularization.

**Results:**

Over a median follow-up of 927 days, 358 patients (20.6%) experienced MACE. The Kaplan-Meier curves showed significant differences in the cumulative incidence of MACE among prespecified groups (*p* < 0.001). Multivariable Cox regression analysis revealed that higher TyG was significantly associated with an increased risk of MACE in patients without HHcy (HR: 2.36, 95% CI: 1.53–3.64, *p* < 0.001), but not in patients with HHcy (HR: 1.31, 95% CI: 0.60–2.87, *p* = 0.503). Restricted cubic splines only demonstrated the prognostic value of TyG in patients without HHcy. A significant interaction was observed for MACE between TyG and HHcy (*p* for interaction = 0.01).

**Conclusions:**

The prognostic value of TyG was modified by HHcy in ACS patients undergoing PCI. Higher TyG was only associated with an increased risk of MACE in ACS patients without HHcy, but not in ACS patients with HHcy.

## 1 Introduction

Coronary artery disease (CAD) remains a leading cause of global mortality and health loss, and causes an increasing public health burden worldwide (1, 2). Despite guideline-recommended medical therapy and percutaneous coronary intervention (PCI), patients with acute coronary syndrome (ACS) still faced relatively high cardiovascular risks (3). Therefore, it is necessary to further identify reliable prognostic factors for ACS patients undergoing PCI.

Insulin resistance (IR) and homocysteine (Hcy) are both metabolic risk factors for CAD. IR refers to decreased responsiveness of the body to insulin, resulting in the inability of insulin to stimulate tissue cells for glucose uptake and utilization. Although the hyperinsulinemic-euglycemic clamp (HEC) is the gold standard for assessing IR (4), it is rarely utilized in clinical practice due to its complexity, time consumption, expense, and invasiveness. Triglyceride glucose (TyG) is a novel index composed of blood lipids and glucose. It is considered as a reliable index reflecting insulin resistance, showing consistent accuracy with the hyperinsulinemic-euglycemic clamp, and is more economical and convenient (5–7). Homocysteine (Hcy) is a sulfhydryl-containing amino acid present in plasma and an important intermediary product in the metabolism of cysteine and methionine. Hyperhomocysteinemia (HHcy) is independently associated with blood lipid levels (8) and promotes IR through several synergistic mechanisms (9–11). Hcy promotes endothelial dysfunction, prothrombotic effects, and irreversible alteration in remodeling of the vessel wall (12–15).

Previous studies have demonstrated that higher TyG is independently associated with poor prognosis in CAD patients (16–19). For decades, HHcy has been recognized as one of the cardiovascular risk factors (20, 21). However, the prognostic value of TyG in ACS patients undergoing PCI and the interaction between TyG and Hcy remain unclear. In this study, we aimed to explore the prognostic value of TyG in ACS patients undergoing PCI and further evaluate the interaction between TyG and HHcy.

## 2 Materials and methods

### 2.1 Study population

**Figure 1:**
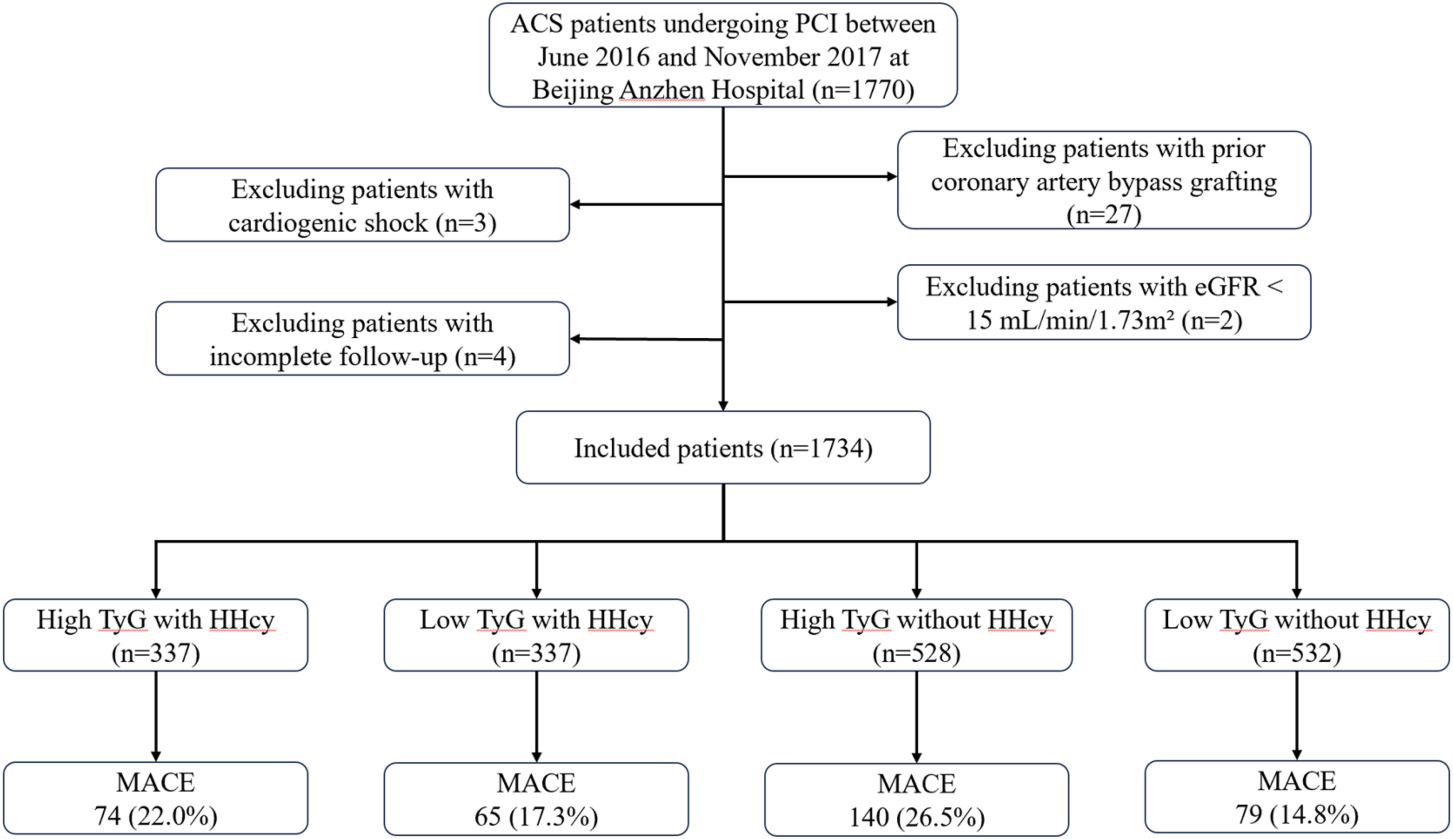
Study flow chart. ACS, acute coronary syndrome; PCI, percutaneous coronary intervention; HHcy, hyperhomocysteinemia; eGFR, estimated glomerular filtration rate; TyG, triglyceride glucose; MACE, major adverse cardiovascular events.

This is a single-center, retrospective analysis including 1,734 ACS patients undergoing PCI between June 2016 and November 2017 at Beijing Anzhen Hospital. The exclusion criteria were as follows: Patients with (1) prior coronary artery bypass grafting; (2) cardiogenic shock; (3) estimated glomerular filtration rate (eGFR) < 15 ml/min/1.73 m^2^; (4) incomplete follow-up (Figure 1). In the final analysis, 1,734 patients were continuously enrolled and categorized into four groups based on HHcy status and the optimal cut-off value of TyG.

The diagnosis of ACS was determined according to the 2013 ACCF/AHA and 2014 AHA/ACC guidelines (22, 23). ST-segment elevation myocardial infarction (STEMI) was defined as myocardial ischemic symptoms accompanied by persistent electrocardiographic ST elevation and elevated levels of creatine kinase or cardiac troponin higher than the upper limit of the normal range. The diagnosis of non-ST-segment elevation myocardial infarction (NSTEMI) and unstable angina (UA) was determined based on the presence or absence of elevated cardiac biomarkers. HHcy was defined as Hcy > 15 μmol/L.

### 2.2 Data collection and definitions

Data on demographics, medical history, procedural results, and medications were collected from the electronic medical records system using standardized questionnaires. Body mass index (BMI) was calculated by weight/height^2^ (kg/m^2^). Fasting blood glucose (FBG), total cholesterol (TC), low-density lipoprotein-cholesterol (LDL-C), high-density lipoprotein-cholesterol (HDL-C), triglyceride (TG), and homocysteine levels were measured after admission in the central laboratory of Beijing Anzhen Hospital. TyG index was calculated using the formula Ln (TG[mg/dl] × FBG[mg/dl]/2). Hypertension was diagnosed when systolic blood pressure was ≥140 mmHg and/or diastolic blood pressure (DBP) was ≥90 mmHg and/or receiving antihypertension treatment. Diabetes was considered when 2-h blood glucose after an oral glucose tolerance test (OGTT) was ≥11.1 mmol/L, and/or FBG was ≥7.0 mmol/L and/or casual plasma glucose was ≥11.1 mmol/L along with diabetic symptoms, and/or taking antidiabetic medication. Chronic kidney disease (CKD) was defined as an estimated glomerular filtration rate (eGFR) ≤60 ml/min/1.73 m^2^. Heart failure (HF) was diagnosed in patients with symptoms/signs of congestive heart failure accompanied by elevated B-type natriuretic peptide (BNP) levels, receiving ongoing therapy for heart failure, or left ventricular ejection fraction (LVEF) ≤ 40%.

### 2.3 Follow-up and endpoints

Follow-up assessments were conducted at one month and subsequently every six months after discharge. Information of adverse events were obtained through telephone contacts and electronic medical records by trained personnel who were blinded to the patients' baseline data. The primary endpoint was major adverse cardiovascular events (MACE), a composite of all-cause death, nonfatal myocardial infarction (MI), nonfatal stroke, and unplanned repeat revascularization (URR). Any non-staged revascularization that occurred after the initial PCI was considered as an unplanned repeat revascularization.

### 2.4 Statistical analysis

Due to the lack of comparable studies evaluating the effect of HHcy on the prognostic value of TyG index, a power analysis to determine the required sample size was waived. Continuous variables with a normal distribution were reported as means ± standard deviations, while those with non-normal distribution were reported as medians and interquartile ranges. Categorical variables were presented as frequencies and percentages. Differences in continuous variables among groups were examined using unpaired *t*-test or Mann–Whitney *U* test. The comparison of categorical variables between groups was performed using the chi-squared test.

Correlation analysis was conducted using Pearson's or Spearman's tests. Survival analyses for MACE were carried out using Kaplan–Meier curves and *p* value was calculated by log-rank test. Cox proportional hazards analysis was used to calculate hazard ratios (HR) and 95% confidence intervals (CI). Variables that showed statistical significance in baseline characteristics, as well as those of clinical importance, were included in the multivariate Cox proportional hazards model. *P* for interaction was tested by adding an interaction term to the Cox proportional hazards model. The linear association and optimal cut-off value of TyG index was evaluated using restricted cubic spline (RCS) with four knots. A two-sided *p* value <0.05 was defined as statistically significant. All statistical analyses were performed using R (version 4.3.2) and SPSS (version 27.0).

## 3 Results

### 3.1 Baseline characteristics

A total of 1,734 patients (mean age 59.8 ± 10.5 years, male 76.6%), 674 (38.9%) of whom had HHcy, were included in this analysis. Baseline characteristics of the study population stratified by HHcy were summarized in Table 1. In comparison to patients with HHcy, patients without HHcy tended to be female, have higher BMI and increased prevalence of diabetes mellitus (DM), but lower proportions of current smoking, HF, CKD, and previous MI. Additionally, patients without HHcy demonstrated higher HDL-C, glycated hemoglobin (HbA1c), and FBG levels, along with lower high-sensitivity C-reactive protein (hsCRP) and serum creatinine (Scr) levels. Moreover, patients without HHcy had lower syntax scores and received a higher proportion of aspirin treatment. TyG index values between groups showed no significant difference.

**Table 1:** Baseline characteristics of the study population stratified by HHcy.

Variables	Overall	Patients without HHcy	Patients with HHcy	*p* value
	*N* = 1,734	*N* = 1,060	*N* = 674	
Demographics				
Age (years)	59.82 ± 10.45	59.57 ± 9.89	60.22 ± 11.27	0.206
Male, *n* (%)	1,329 (76.6)	747 (70.5)	582 (86.4)	<0.001
BMI (kg/m^2^)	25.68 ± 3.09	25.52 ± 3.02	25.92 ± 3.19	0.008
Medical history				
Current smoking, *n* (%)	766 (44.2)	419 (39.5)	347 (51.5)	<0.001
Hypertension, *n* (%)	1,106 (63.8)	678 (64.0)	428 (63.5)	0.886
DM, *n* (%)	799 (46.1)	543 (51.2)	256 (38.0)	<0.001
HF, *n* (%)	125 (7.2)	50 (4.7)	75 (11.1)	<0.001
CKD, *n* (%)	106 (6.1)	41 (3.9)	65 (9.6)	<0.001
Previous MI, *n* (%)	333 (19.2)	174 (16.4)	159 (23.6)	<0.001
Previous PCI, *n* (%)	343 (19.8)	206 (19.4)	137 (20.3)	0.694
Laboratory measurements				
TC (mmol/L)	4.15 ± 0.99	4.17 ± 0.99	4.10 ± 0.98	0.145
TG (mmol/L)	1.45 (1.01–2.06)	1.43 (1.00–2.01)	1.50 (1.04–2.13)	0.152
LDL-C (mmol/L)	2.44 ± 0.81	2.44 ± 0.81	2.44 ± 0.81	0.925
HDL-C (mmol/L)	1.03 ± 0.23	1.05 ± 0.24	1.00 ± 0.23	<0.001
hsCRP (mg/L)	1.36 (0.65–3.48)	1.23 (0.58–2.97)	1.71 (0.73–4.69)	<0.001
Hcy (*μ*mol/L)	13.20 (10.10–18.20)	10.75 (9.10–12.90)	19.70 (17.00–24.30)	<0.001
HbA1c (%)	6.1 (5.6–7.1)	6.3 (5.6–7.3)	5.9 (5.5–6.8)	<0.001
FBG (mmol/L)	5.78 (5.23–6.94)	5.92 (5.24–7.16)	5.67 (5.21–6.70)	<0.001
Scr (μmol/L)	70.30 (62.23–79.70)	68.20 (60.30–75.62)	74.30 (66.75–86.60)	<0.001
LVEF (%)	65 (60–68)	65 (60–68)	64 (58–68)	0.001
Clinical diagnosis				
STEMI, *n* (%)	227 (13.1)	109 (10.3)	118 (17.5)	<0.001
NSTEMI, *n* (%)	222 (12.8)	115 (10.8)	107 (15.9)	0.003
UA, *n* (%)	1,285 (74.1)	836 (78.9)	449 (66.6)	<0.001
Procedural results				
DES, *n* (%)	1,425 (82.2)	867 (81.8)	558 (82.8)	0.642
BRS, *n* (%)	98 (5.7)	55 (5.2)	43 (6.4)	0.347
DCB, *n* (%)	111 (6.4)	72 (6.8)	39 (5.8)	0.463
SYNTAX score	21.23 ± 10.90	20.79 ± 10.74	21.92 ± 11.13	0.035
Complete recascularization, *n* (%)	1,063 (61.3)	649 (61.2)	414 (61.4)	0.975
Medications at discharge				
Asprin, *n* (%)	1,718 (99.1)	1,057 (99.7)	661 (98.1)	0.001
P2Y12, *n* (%)	1,732 (99.9)	1,058 (99.8)	674 (100.0)	0.687
Statins, *n* (%)	1,734 (100.0)	1,060 (100.0)	674 (100.0)	0.999
ACEI/ARB, *n* (%)	1,217 (70.2)	742 (70.0)	475 (70.5)	0.875
*β*-blocker, *n* (%)	836 (48.2)	491 (46.3)	345 (51.2)	0.054
TyG	8.90 ± 0.61	8.91 ± 0.63	8.88 ± 0.58	0.296

HHcy, hyperhomocysteinemia; BMI, body mass index; DM, diabetes mellitus; HF, heart failure; CKD, chronic kidney disease; UA, Unstable angina; NSTEMI, Non ST-segment elevation myocardial infarction; STEMI, ST-segment elevation myocardial infarction; TC, Total cholesterol; TG, Triglyceride; LDL-C, Low-density lipoprotein-cholesterol; HDL-C, High-density lipoprotein-cholesterol; hsCRP, high-sensitivity C-reactive protein; Hcy, homocysteine; HbA1c, glycated hemoglobin; FBG, Fasting blood glucose; Scr, serum creatinine; LVEF, left ventricular ejection fractions; ACEI, angiotensin converting enzyme inhibitors; ARB, angiotensin II receptor blockers; DCB, drug-coated balloon; DES, drug-eluting stent; BRS, bioresorbable scaffold.

The optimal cut-off value of TyG index for MACE was 8.86 (Figure 5A). Baseline characteristics of the study population according to HHcy and the optimal cut-off value of TyG index were shown in Table 2. Regardless of HHcy, patients with high TyG were more likely to be younger, have lower BMI, a history of DM and lower HDL-C levels, but higher levels of TC, TG, LDL-C, HbA1c, FBG, and syntax scores. Additionally, in patients without HHcy, those with high TyG were more likely to be current smokers, have higher levels of hsCRP, and be more frequently treated with β-blockers.

**Table 2:** Baseline characteristics of the study population according to HHcy and the optimal cut-off value of TyG index.

Variables	Patients without HHcy		*p* value	Patients with HHcy		*p* value
	High TyG *N* = 528	Low TyG *N* = 532		High TyG *N* = 337	Low TyG *N* = 337	
Demographics						
Age (years)	58.30 ± 10.22	60.82 ± 9.39	<0.001	57.95 ± 12.17	62.49 ± 9.79	<0.001
Male, *n* (%)	375 (71.0)	372 (69.9)	0.746	291 (86.4)	291 (86.4)	>0.999
BMI (kg/m^2^)	26.03 ± 3.28	25.01 ± 2.64	<0.001	26.41 ± 3.02	25.43 ± 3.29	<0.001
Medical history						
Current smoking, *n* (%)	239 (45.3)	180 (33.8)	<0.001	182 (54.0)	165 (49.0)	0.218
Hypertension, *n* (%)	340 (64.4)	338 (63.5)	0.820	225 (66.8)	203 (60.2)	0.093
DM, *n* (%)	344 (65.2)	199 (37.4)	<0.001	155 (46.0)	101 (30.0)	<0.001
HF, *n* (%)	27 (5.1)	23 (4.3)	0.644	32 (9.5)	43 (12.8)	0.221
CKD, *n* (%)	21 (4.0)	20 (3.8)	0.980	29 (8.6)	36 (10.7)	0.434
Previous MI, *n* (%)	93 (17.6)	81 (15.2)	0.334	69 (20.5)	90 (26.7)	0.07
Previous PCI, *n* (%)	106 (20.1)	100 (18.8)	0.654	69 (20.5)	68 (20.2)	>0.999
Laboratory measurements						
TC (mmol/L)	4.39 ± 1.00	3.97 ± 0.94	<0.001	4.42 ± 1.02	3.79 ± 0.82	<0.001
TG (mmol/L)	2.01 (1.64–2.68)	1.02 (0.82–1.28)	<0.001	2.13 (1.75–2.71)	1.05 (0.85–1.30)	<0.001
LDL-C (mmol/L)	2.54 ± 0.80	2.34 ± 0.80	<0.001	2.63 ± 0.83	2.26 ± 0.75	<0.001
HDL-C (mmol/L)	0.98 ± 0.19	1.13 ± 0.26	<0.001	0.95 ± 0.20	1.05 ± 0.24	<0.001
hsCRP (mg/L)	1.46 (0.74–3.35)	1.02 (0.45–2.37)	<0.001	1.66 (0.73–5.31)	1.78 (0.73–3.84)	0.461
Hcy (μmol/L)	10.55 (9.00–12.60)	11.00 (9.20–13.10)	0.250	19.30 (16.80–24.50)	20.00 (17.10–24.10)	0.466
HbA1c (%)	6.80 (5.90–7.90)	5.80 (5.40–6.70)	<0.001	6.00 (5.60–7.20)	5.80 (5.50–6.40)	<0.001
FBG (mmol/L)	6.69 (5.69–8.12)	5.54 (5.11–6.12)	<0.001	6.01 (5.32–7.37)	5.45 (5.10–6.08)	<0.001
Scr (μmol/L)	68.45 (60.77–77.45)	67.30 (60.27–74.62)	0.051	75.10 (67.30–87.30)	73.60 (65.90–85.50)	0.189
LVEF (%)	65.00 (60.00–68.00)	65.00 (60.00–68.00)	0.965	64.00 (58.00–68.00)	63.00 (58.00–67.00)	0.252
Clinical diagnosis						
STEMI, *n* (%)	62 (11.7)	47 (8.8)	0.145	62 (18.4)	56 (16.6)	0.612
NSTEMI, *n* (%)	69 (13.1)	46 (8.6)	0.027	59 (17.5)	48 (14.2)	0.292
UA, *n* (%)	397 (75.2)	439 (82.5)	0.004	216 (64.1)	233 (69.1)	0.191
Procedural results						
DES, *n* (%)	428 (81.1)	439 (82.5)	0.592	280 (83.1)	278 (82.5)	0.919
BRS, *n* (%)	22 (4.2)	33 (6.2)	0.175	21 (6.2)	22 (6.5)	>0.999
DCB, *n* (%)	42 (8.0)	30 (5.6)	0.169	22 (6.5)	17 (5.0)	0.509
SYNTAX score	21.59 ± 10.35	20.00 ± 11.06	0.015	22.84 ± 11.32	21.01 ± 10.88	0.033
Complete revascularization, *n* (%)	311 (58.9)	338 (63.5)	0.138	193 (57.3)	221 (65.6)	0.033
Medications at discharge						
Aspirin, *n* (%)	525 (99.4)	532 (100.0)	0.245	331 (98.2)	330 (97.9)	>0.999
P2Y12, *n* (%)	528 (100.0)	530 (99.6)	0.482	337 (100.0)	337 (100.0)	>0.999
Statins, *n* (%)	528 (100.0)	532 (100.0)	0.999	337 (100.0)	337 (100.0)	>0.999
ACEI/ARB, *n* (%)	372 (70.5)	370 (69.5)	0.799	245 (72.7)	230 (68.2)	0.237
β-blocker, *n* (%)	262 (49.6)	229 (43.0)	0.037	181 (53.7)	164 (48.7)	0.218
TyG	9.40 ± 0.44	8.42 ± 0.33	<0.001	9.33 ± 0.40	8.42 ± 0.32	<0.001

All abbreviations as in Table 1.

### 3.2 Correlation analysis between TyG and other variables

**Figure 2:**
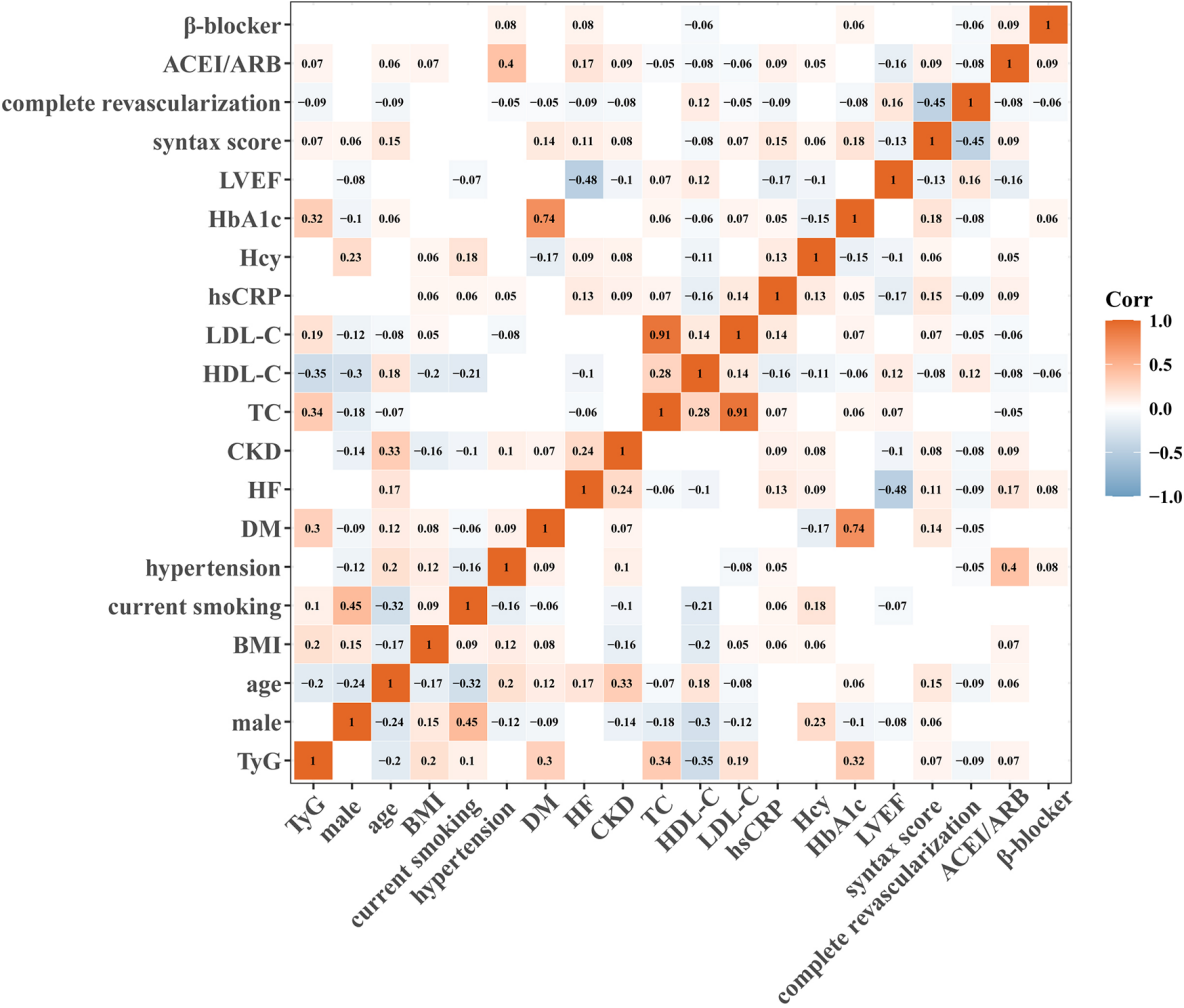
Correlation analysis of baseline characteristics.

Figure 2 shows the correlation analysis between TyG and clinical risk factors. TyG showed a weak but significant positive correlation with BMI, current smoking, DM, TC, LDL-C, HbA1c, syntax score, and ACEI/ARB at discharge, while negatively correlating with age, HDL-C, and complete revascularization (all *p* < 0.05).

### 3.3 Prognostic value of TyG in patients with or without HHcy

**Figure 3:**
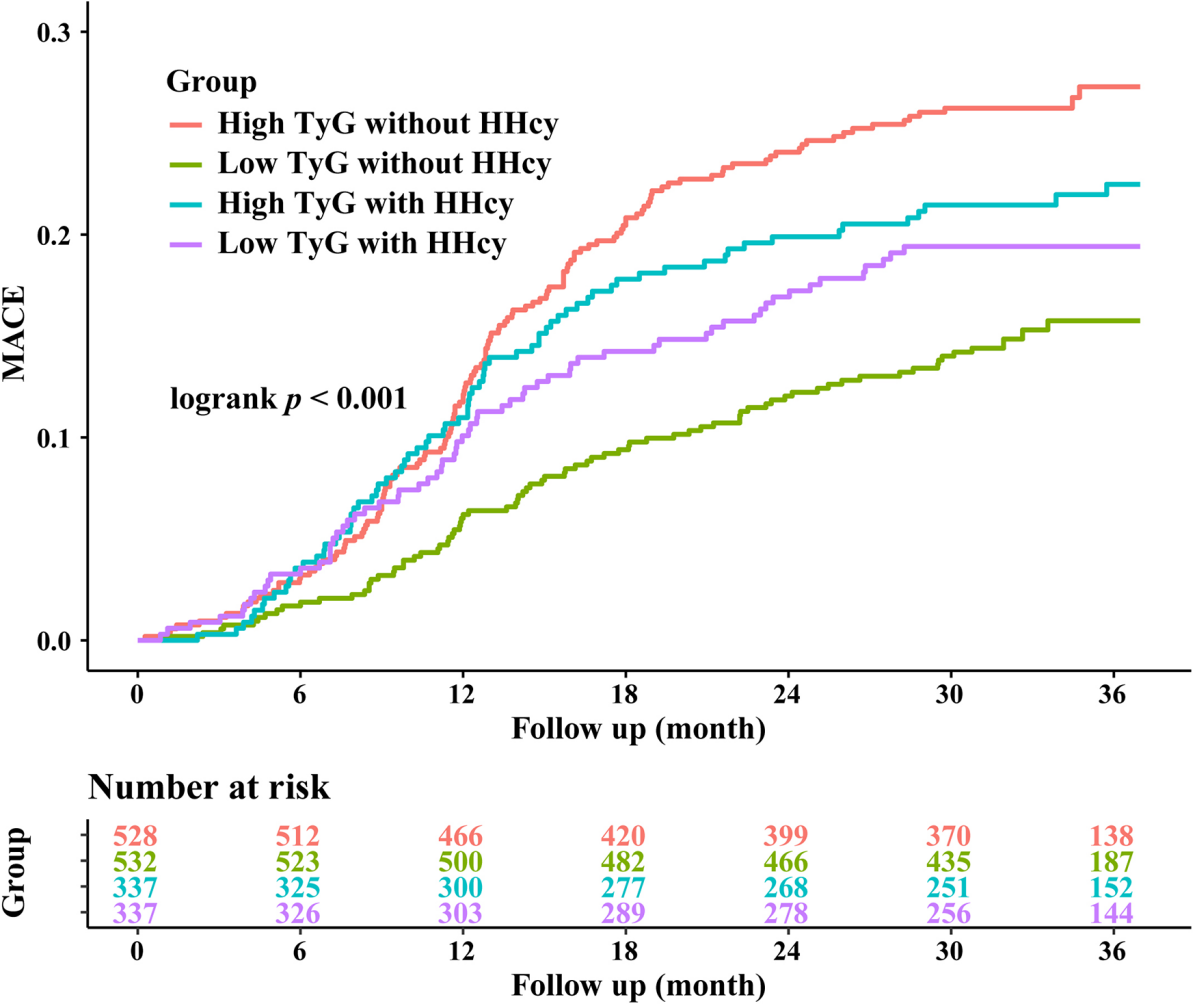
Kaplan-Meier curves for MACE stratified by HHcy and TyG index.

**Figure 4:**
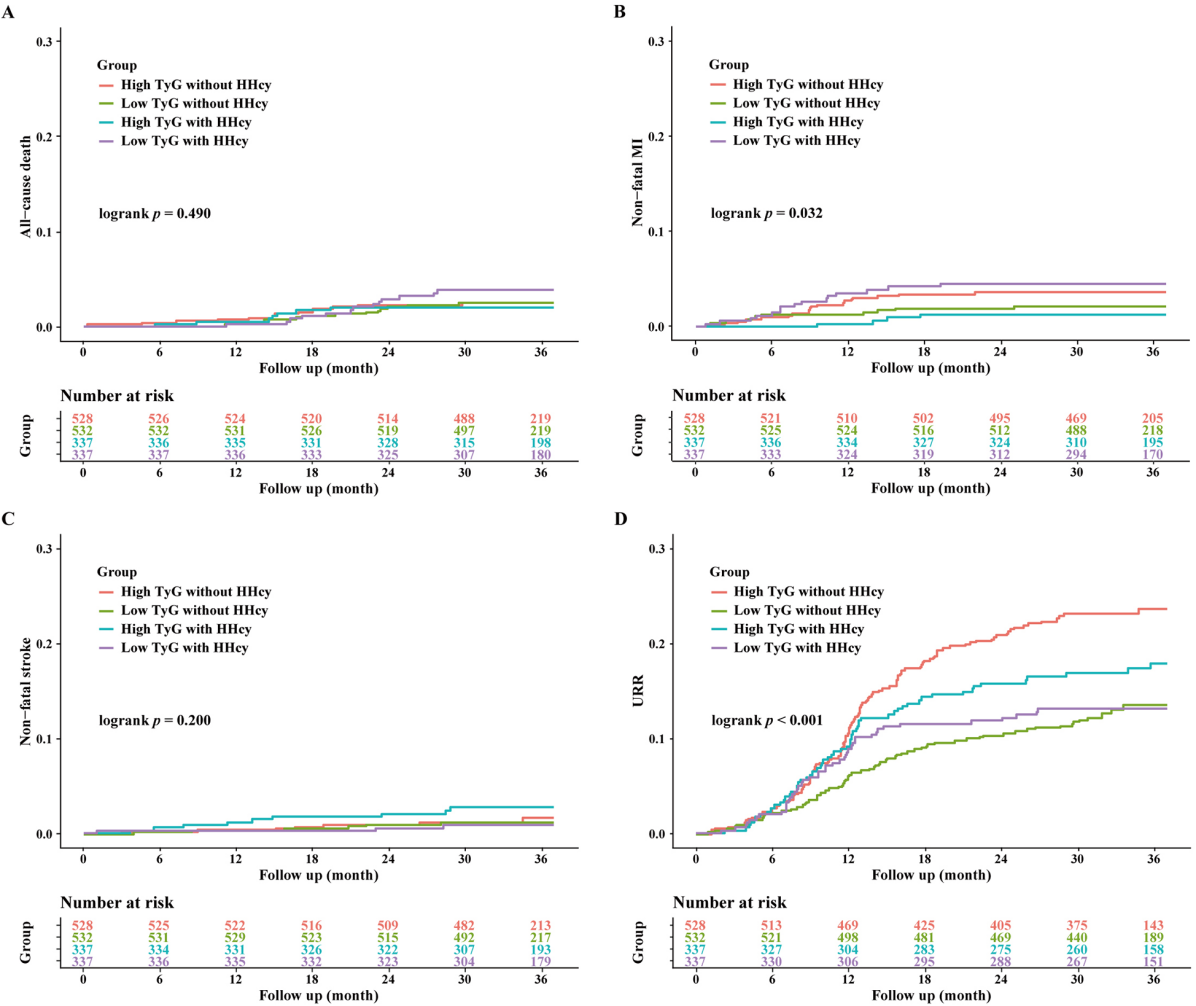
Kaplan-Meier curves for the secondary endpoint stratified by HHcy and TyG index. **(A)** Kaplan-Meier curves for all-cause death; **(B)** Kaplan-Meier curves for nonfatal MI; **(C)** Kaplan-Meier curves for nonfatal stroke; **(D)** Kaplan-Meier curves for URR.

Over a median follow-up of 927 days, 358 patients (20.6%) experienced MACE. Detailed information of MACE was described in Table 3. The Kaplan–Meier curves showed significant differences in cumulative incidence of MACE among prespecified groups (logrank *p* < 0.001, Figure 3). This difference was primarily driven by unplanned repeat revascularization (logrank *p* < 0.001, Figure 4). A significant interaction was observed between TyG and HHcy (*p* for interaction = 0.01). Multivariable Cox regression analysis revealed that higher TyG was significantly associated with an increased risk of MACE in patients without HHcy (Table 4) (Model 1: HR: 2.02, 95% CI: 1.62–2.47, *p* < 0.001; Model 2: HR: 2.18, 95% CI: 1.79–2.64, *p* < 0.001; Model 3: HR: 2.00, 95% CI: 1.67–2.47, *p* < 0.001; Model 4: HR: 2.39, 95% CI: 1.58–3.63, *p* < 0.001; Model 5: HR: 2.38, 95% CI: 1.55–3.65, *p* < 0.001; Model 6: HR: 2.36, 95% CI: 1.53–3.64, *p* < 0.001), but failed to demonstrate similar prognostic value in patients with HHcy (Table 4) (Model 1: HR: 1.28, 95% CI: 0.96–1.70, *p* = 0.016; Model 2: HR: 1.44, 95% CI: 1.08–1.93, *p* = 0.014; Model 3: HR: 1.37, 95% CI: 0.99–1.87, *p* = 0.052; Model 4: HR: 1.58, 95% CI: 0.73–3.42, *p* = 0.243; Model 5: HR: 1.27, 95% CI: 0.59–2.74, *p* = 0.542; Model 6: HR: 1.31, 95% CI: 0.60–2.87, *p* = 0.504). In the overall study population, Hcy was not associated with the risk of MACE (HR: 1.00, 95% CI: 0.99–1.02, *p* = 0.634).

**Table 3:** Adverse cardiovascular events stratified by HHcy and the optimal cut-off value of TyG index.

Adverse cardiovascular disease	Patients without HHcy (*N* = 1,060)		*p* value	Patients with HHcy (*N* = 674)		*p* value
	High TyG	Low TyG		High TyG	Low TyG	
MACE, *n* (%)	140 (26.5)	79 (14.8)	<0.001	74 (22.0)	65 (17.3)	0.392
All-cause death, *n* (%)	13 (2.5)	13 (2.4)	0.984	7 (2.1)	13 (3.9)	0.173
MI, *n* (%)	19 (3.6)	11 (2.1)	0.133	4 (1.2)	15 (4.5)	0.010
Stroke, *n* (%)	7 (1.3)	6 (1.1)	0.770	9 (2.7)	3 (0.9)	0.081
URR, *n* (%)	121 (22.9)	67 (12.6)	<0.001	58 (17.2)	44 (13.1)	0.132

MACE, major adverse cardiovascular event; MI, myocardial infarction; URR, unplanned repeat revascularization.

**Table 4:** Univariate and multivariate Cox proportional hazard analyses for MACE stratified by HHcy.

	TyG index as a continuous variable			
	Patients without HHcy (HR 95%CI)	*p* value	Patients with HHcy (HR 95%CI)	*p* value
Model 1	2.015 (1.673–2.427)	<0.001	1.277 (0.960–1.698)	0.016
Model 2	2.175 (1.790–2.643)	<0.001	1.443 (1.078–1.929)	0.014
Model 3	1.998 (1.618–2.468)	<0.001	1.366 (0.998–1.869)	0.052
Model 4	2.393 (1.578–3.628)	<0.001	1.583 (0.732–3.420)	0.243
Model 5	2.379 (1.552–3.648)	<0.001	1.270 (0.589–2.741)	0.542
Model 6	2.359 (1.528–3.640)	<0.001	1.308 (0.596–2.870)	0.504

Model 1: Unadjusted.

Model 2: Adjusted for age, sex, BMI.

Model 3: Model 2 + current smoking, hypertension, DM, HF, CKD.

Model 4: Model 3 + LDL-C, HDL-C, TG, hs-CRP.

Model 5: Model 4 + SYNTAX score + complete revascularization.

Model 6: Model 5 + β blocker at discharge.

**Figure 5:**
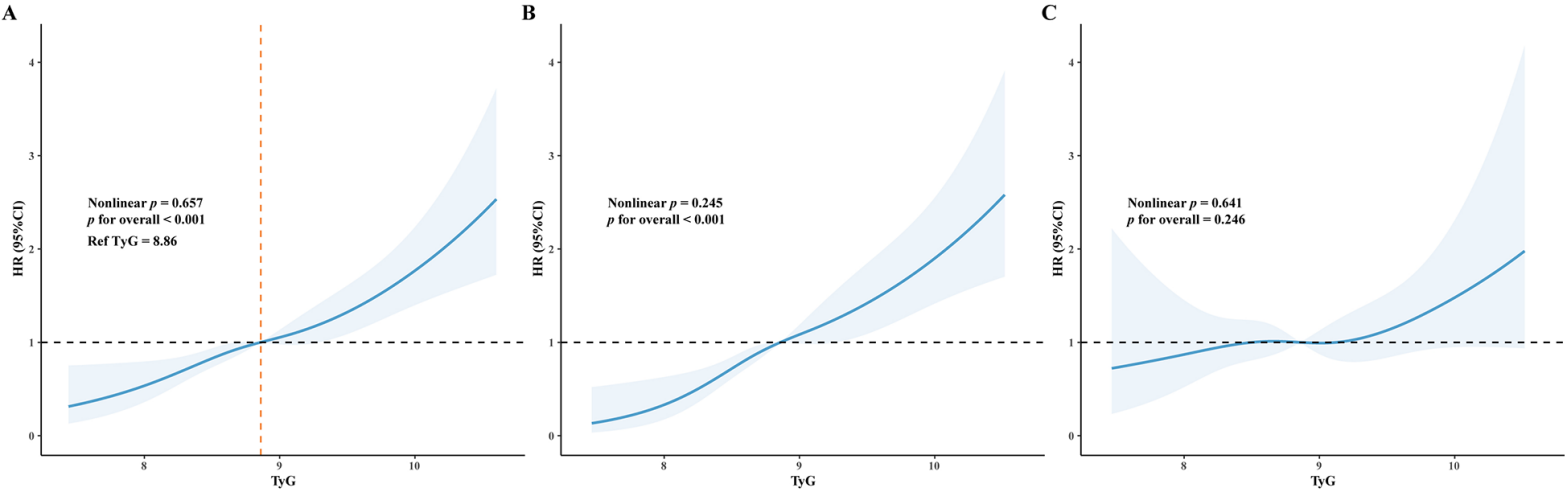
Restricted cubic splines (RCS) curve demonstrated the association between TyG index and MACE. The blue line represents the hazard ratio (HR), and the shaded area represents the 95% confidence interval (CI). **(A)** RCS curve for all patients; (**B**) RCS curve for patients without HHcy; **(C)** RCS curve for patients with HHcy.

RCS curves were used to visualize the linear association between TyG and MACE (Figure 5). In the overall ACS population, a linear association was observed between higher TyG and increased MACE risk (*p* for overall <0.001, *p* for nonlinear = 0.657). Among patients without HHcy, high TyG was linearly associated with an increased MACE risk (*p* for overall <0.001, *p* for nonlinear = 0.245). By contrast, among patients with HHcy, TyG was not associated with MACE risk (*p* for overall = 0.246).

### 3.4 Impact of HHcy on the prognostic value of TyG

**Figure 6:**
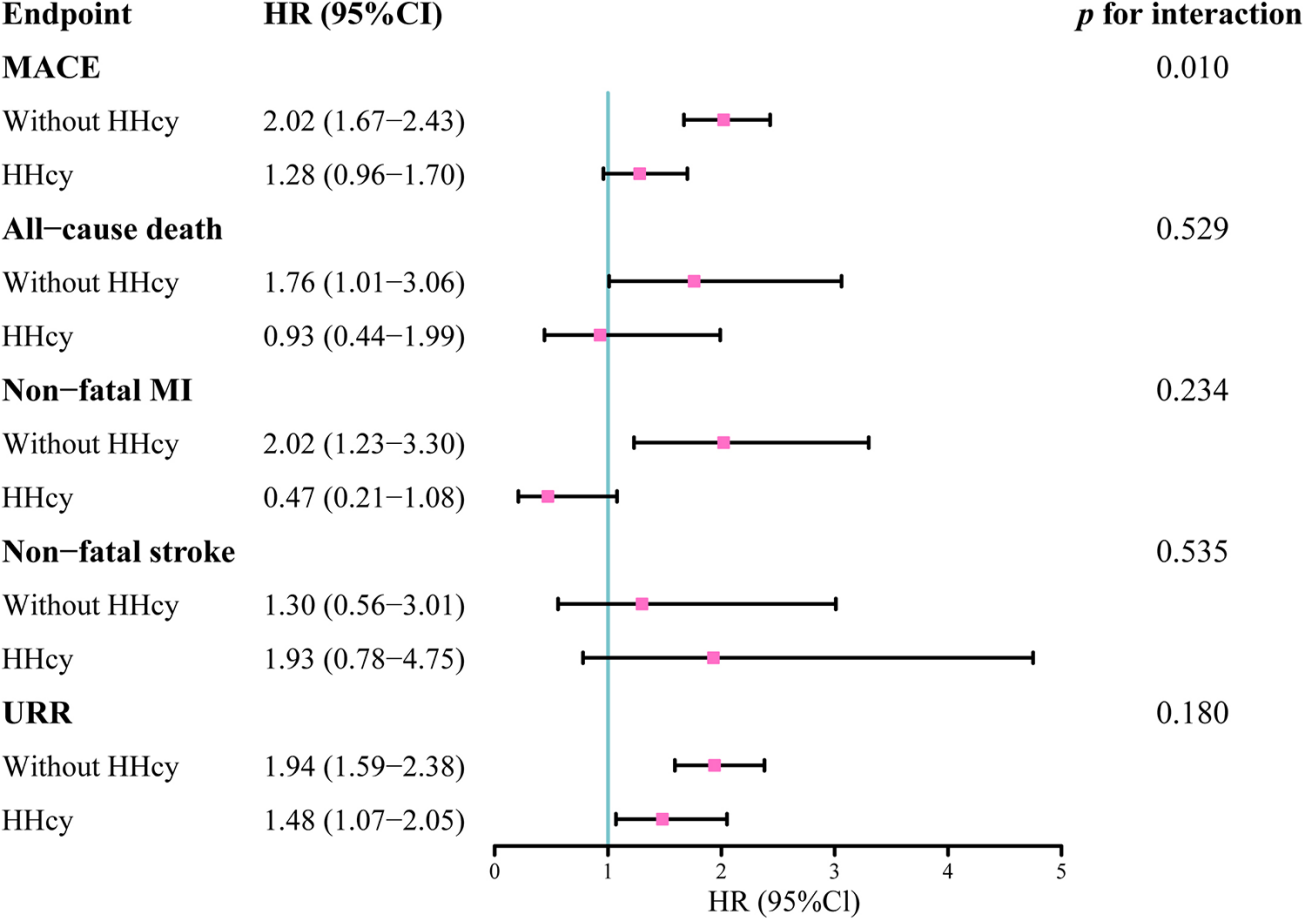
Hazard ratios of TyG index as a continuous variable on the endpoints in patients with and without HHcy.

Figure 6 presented the impact of HHcy on the prognostic value of TyG for MACE, all-cause death, nonfatal MI, nonfatal stroke, and unplanned repeat revascularization. A significant interaction between HHcy and TyG in predicting MACE was observed.

## 4 Discussion

Based on our knowledge, the present study is the first to evaluate the prognostic value of TyG in patients with ACS undergoing PCI, and further investigate the interaction between TyG and HHcy. Our analysis yielded the following findings: (1) Regardless of continuous variable or categorical variable, TyG was independently associated with poorer prognosis in ACS patients without HHcy. (2) A linear relationship was observed between TyG and MACE. [3] The prognostic value of TyG was demonstrated only in patients without HHcy, but not in patients with HHcy. A significant interaction was observed between TyG and HHcy.

As early as 1969, McCully proposed the association between homocysteine and atherosclerosis (24). The vascular pathology and prognostic value of Hcy have been discussed for decades. Hcy contributes to increased blood coagulation and decreased fibrinolysis through synergistic mechanisms (25). Hcy also induces endothelial dysfunction by promoting oxidative stress, inflammation, elevated production of nitric oxide, hydrogen sulfide (H2S), adenosine, and oxidized low-density lipoprotein (LDL) (13, 14, 26–29). Furthermore, elevated Hcy levels lead to irreversible remodeling of the vessel wall, characterized by increased fibrosis, homocysteinylation of extracellular matrix proteins, and breaks in elastin (14, 15). Consequently, elevated Hcy is reasonably recognized as a risk factor for CVD. However, the effect of HHcy on cardiovascular disease remained controversial in clinic practice. Meta-analysis of observational studies indicated that elevated Hcy was associated with an increased risk of cardiovascular risk in healthy populations (20) and ACS patients (21). But it is worth noting that lowering Hcy levels through diet or medical therapy did not consistently reduce the risk of CVD (30). An early meta-analysis in 2010 showed that folic acid supplementation could reduce Hcy levels by 25%, yet it did not lower the risk of cardiovascular events and all-cause mortality (31). Another meta-analysis of 30 randomized controlled trials indicated a 10% lower risk of stroke and a 4% reduction in overall CVD risk by folic acid supplementation (32). Cochrane review in 2017 demonstrated that B-vitamins supplementation slightly reduced the risk of stroke in patients without end-stage renal disease, but did not reduce the risk of MI or all-cause mortality (33). Therefore, a deeper understanding of the relationship between HHcy and cardiovascular diseases is still required.

The association between IR and adverse cardiovascular outcomes has been widely acknowledged. While the hyperinsulinemic-euglycemic clamp (HEC) is regarded as the gold standard for IR assessment, its clinical utility is limited due to complexity, time consumption, expense, and the need for frequent blood tests. The TyG index provides high sensitivity and specificity in measuring insulin resistance, with the added advantages of convenience and cost-effectiveness, making it more suitable for clinical application (5–7). The prognostic value of TyG in ACS patients has been confirmed by previous studies (16, 19, 34–40). A meta-analysis including 13,684 ACS patients showed that the highest category of TyG was associated with twofold increased MACE risk compared to the lowest category (34). Liang SC et al. analyzed data of CAD patients from 41 studies and concluded that patients with higher TyG levels were at a higher risk of CAD, with more severe coronary artery lesions, and suffered poorer prognosis compared to those with lower TyG levels (19). A cohort study included 1,158 Chinese patients and reported that higher TyG was associated with worse prognosis in ACS patients with prior coronary artery bypass grafting undergoing PCI (16). Wang L et al. enrolled 2,531 DM patients undergoing coronary angiography for ACS and found that TyG could predict MACE risk in ACS patients with DM independently of known cardiovascular risk factors (35). Subgroup analyses in these studies generally incorporated clinical risk factors such as age, gender, hypertension, and diabetes, but never took the influence of HHcy into consideration. In our study, we found consistent results with previous evidence that higher TyG was associated with an increased risk of MACE. Moreover, serum Hcy levels showed no correlation with MACE risk. Importantly, a significant interaction between TyG and hcy was observed. TyG was only associated with increased MACE risk in ACS patients without HHcy, but not in ACS patients with HHcy. Therefore, we reasonably assumed that the prognostic value of TyG was modified by HHcy.

The underlying mechanisms behind these findings remain unclear and need further investigation. HHcy promotes insulin resistance through various synergistic mechanisms such as protein cysteine-homocysteinylation (9), MDM2-mediated ubiquitination of HSF1 (10), and modulation of M2 macrophage polarization via estrogen suppression (11). Additionally, supplementation with folic acid and vitamin B12 has been shown to improve insulin resistance in intrauterine growth retardation rats (41). Plasma homocysteine levels were also found to be independently associated with conventional atherogenic lipid profiles and remnant cholesterol in adults (8). These mechanisms may be relevant to the interaction between HHcy and TyG observed in our analysis.

In our study, the gender distribution differed between the HHcy and non-HHcy groups, with a relatively low proportion of females in the overall population. However, after adjusting for gender in the multivariable Cox regression analysis, the discrepancy in the prognostic value of the TyG index remained. To further elucidate potential sexual dimorphisms in the prognostic value of the TyG index, we analyzed and reported the baseline characteristics and prognostic value of TyG separately for males and females in both HHcy and non-HHcy groups (Supplementary Tables S1 and S2). Our findings suggest that, among patients with HHcy, gender appears to influence the prognostic value of the TyG index. However, possibly due to the limited sample size, we did not observe a significant interaction between TyG and gender (*p* for interaction = 0.069). Future studies should include a larger sample size and higher proportion of female participants to further validate and extend our conclusions.

The present study has several limitations. Firstly, this is a single-center, retrospective analysis of a prospective registry. Some confounding factors, such as unhealthy lifestyles and obstructive sleep apnea syndrome, could influence the results. Secondly, the analysis only relied on fasting TyG index and Hcy levels upon admission, without dynamic monitoring of TyG and Hcy. Thus, fluctuations in TyG and Hcy levels were not taken into consideration. Thirdly, the study did not document the prescription of homocysteine-lowering medications such as folic acid and vitamin B, which could potentially affect Hcy levels. Fourth, due to the lack of comparable studies evaluating the effect of HHcy on the prognostic value of TyG, a power analysis to determine the required sample size was waived. Given the substantial sample size of 1,734 patients, we believe our findings are acceptable and provide valuable insights into this area. Finally, the study population consisted entirely of Chinese, and external validation was not conducted. Therefore, the general applications of this finding should be made with caution.

## 5 Conclusion

The prognostic value of TyG was modified by HHcy in ACS patients undergoing PCI. Higher TyG was only associated with an increased risk of MACE in ACS patients without HHcy, but not in ACS patients with HHcy.

## Data Availability

The original contributions presented in the study are included in the article/Supplementary Material, further inquiries can be directed to the corresponding authors.
