# Supplementary material for: Effect of hyperhomocysteinemia on the prognostic value of triglyceride glucose index in patients with acute coronary syndrome
Source: Front Cardiovasc Med. 2025 Jan 10;11:1517437. doi: 10.3389/fcvm.2024.1517437 (PMC11757877; doi:10.3389/fcvm.2024.1517437)
Supplement: Supplementary file 1 [file Table1.docx]

**Table S1** Baseline characteristics of the study population stratified by gender identity

| **Variables** | **Overall** | **Female** | **Male** | ***p* value** |
| --- | --- | --- | --- | --- |
|  | **N=1734** | **N=405** | **N=1329** |  |
| **Demographics** |  |  |  |  |
| Age (years) | 59.82 (10.45) | 64.34 (8.97) | 58.44 (10.48) | <0.001 |
| BMI (kg/m^2^) | 25.68 (3.09) | 24.83 (3.30) | 25.93 (2.98) | <0.001 |
| **Medical history** |  |  |  |  |
| Current smoking, n (%) | 766 (44.2) | 14 (3.5) | 752 (56.6) | <0.001 |
| Hypertension, n (%) | 1106 (63.8) | 300 (74.1) | 806 (60.6) | <0.001 |
| DM, n (%) | 799 (46.1) | 218 (53.8) | 581 (43.7) | <0.001 |
| HF, n (%) | 125 (7.2) | 27 (6.7) | 98 (7.4) | 0.71 |
| CKD, n (%) | 106 (6.1) | 49 (12.1) | 57 (4.3) | <0.001 |
| Previous MI, n (%) | 333 (19.2) | 56 (13.8) | 277 (20.8) | 0.002 |
| Previous PCI, n (%) | 343 (19.8) | 61 (15.1) | 282 (21.2) | 0.008 |
| **Laboratory measurements** |  |  |  |  |
| TC (mmol/L) | 4.15 (0.99) | 4.47 (1.07) | 4.05 (0.94) | <0.001 |
| TG (mmol/L) | 1.45 [1.01, 2.06] | 1.44 [0.99, 1.98] | 1.45 [1.03, 2.10] | 0.331 |
| LDL-C (mmol/L) | 2.44 (0.81) | 2.61 (0.88) | 2.39 (0.78) | <0.001 |
| HDL-C (mmol/L) | 1.03 (0.23) | 1.16 (0.23) | 0.99 (0.22) | <0.001 |
| hsCRP (mg/L) | 1.36 [0.65, 3.48] | 1.58 [0.64, 3.36] | 1.32 [0.65, 3.56] | 0.37 |
| Hcy (μmol/L) | 13.20 [10.10, 18.20] | 11.20 [8.90, 14.30] | 14.10 [10.60, 19.30] | <0.001 |
| HbA1c (%) | 6.10 [5.60, 7.10] | 6.40 [5.70, 7.50] | 6.00 [5.60, 7.00] | <0.001 |
| FBG (mmol/L) | 5.78 [5.23, 6.94] | 5.96 [5.34, 7.10] | 5.74 [5.20, 6.88] | 0.001 |
| Scr (μmol/L) | 70.30 [62.23, 79.70] | 59.60 [53.40, 67.30] | 72.70 [66.00, 82.00] | <0.001 |
| LVEF (%) | 65.00 [60.00, 68.00] | 65.00 [60.00, 69.00] | 64.00 [60.00, 68.00] | 0.006 |
| **Clinical diagnosis** |  |  |  |  |
| STEMI, n (%) | 227 (13.1) | 18 (4.4) | 209 (15.7) | <0.001 |
| NSTEMI, n (%) | 222 (12.8) | 50 (12.3) | 172 (12.9) | 0.818 |
| UA, n (%) | 1285 (74.1) | 337 (83.2) | 948 (71.3) | <0.001 |
| **Procedural results** |  |  |  |  |
| DES, n (%) | 1425 (82.2) | 328 (81.0) | 1097 (82.5) | 0.521 |
| BRS, n (%) | 98 (5.7) | 31 (7.7) | 67 (5.0) | 0.061 |
| DCB, n (%) | 111 (6.4) | 23 (5.7) | 88 (6.6) | 0.574 |
| SYNTAX score | 21.23 (10.90) | 20.08 (9.96) | 21.58 (11.15) | 0.015 |
| Complete  recascularization, n (%) | 1063 (61.3) | 263 (64.9) | 800 (60.2) | 0.097 |
| **Medications at discharge** |  |  |  |  |
| Asprin, n (%) | 1718 (99.1) | 401 (99.0) | 1317 (99.1) | >0.999 |
| P2Y12, n (%) | 1732 (99.9) | 405 (100.0) | 1327 (99.8) | >0.999 |
| Statins, n (%) | 1734 (100.0) | 405 (100.0) | 1329 (100.0) | >0.999 |
| ACEI/ARB, n (%) | 1217 (70.2) | 293 (72.3) | 924 (69.5) | 0.306 |
| β-blocker, n (%) | 836 (48.2) | 203 (50.1) | 633 (47.6) | 0.411 |
| **TyG** | 8.90 (0.61) | 8.90 (0.60) | 8.90 (0.61) | 0.854 |

HHcy, hyperhomocysteinemia; BMI, body mass index; DM, diabetes mellitus; HF, heart failure; CKD, chronic kidney disease; UA, Unstable angina; NSTEMI, Non ST-segment elevation myocardial infarction; STEMI, ST-segment elevation myocardial infarction; TC, Total cholesterol; TG, Triglyceride; LDL-C, Low-density lipoprotein-cholesterol; HDL-C, High-density lipoprotein-cholesterol; hsCRP, high-sensitivity C-reactive protein; Hcy, homocysteine; HbA1c, glycated hemoglobin; FBG, Fasting blood glucose; Scr, serum creatinine; LVEF, left ventricular ejection fractions; ACEI, angiotensin converting enzyme inhibitors; ARB, angiotensin II receptor blockers; DCB, drug-coated balloon; DES, drug-eluting stent; BRS, bioresorbable scaffold

**Table S2** Univariate Cox proportional hazard analyses for MACE stratified by HHcy and gender identity

| **TyG index as a continuous variable** | | | | | | | | |
| --- | --- | --- | --- | --- | --- | --- | --- | --- |
|  | **Male patients without HHcy**  **(HR 95%CI)** | ***p* value** | **Female patients without HHcy**  **(HR 95%CI)** | ***p* value** | **Male patients with HHcy**  **(HR 95%CI)** | ***p* value** | **Female patients with HHcy**  **(HR 95%CI)** | ***p* value** |
| TyG | 1.905 (1.535-2.366) | <0.001 | 2.445 (1.656-3.610) | <0.001 | 1.162 (0.857-1.576) | 0.333 | 2.455 (1.084-5.559) | 0.031 |
